# Quantifying climatic suitability for tourism in Southwest Indian Ocean Tropical Islands: Applying the Holiday Climate Index to Réunion Island

**DOI:** 10.1007/s00484-024-02700-x

**Published:** 2024-05-14

**Authors:** Ariel S. Prinsloo, Jennifer M. Fitchett

**Affiliations:** School of Geography, Archaeology and Environmental Studies, University of the Witwatersrand, Johannesburg, South Africa

**Keywords:** Holiday Climate Index (HCI), Climatic suitability, Urban and beach tourism, SWIO, Réunion Island

## Abstract

Climate is a key resource for tourists and tourism providers. Varied approaches to quantifying climate resources for tourism have been developed in the last 40 years, computing indices from a range of meteorological variables to measure the comparative climatic suitability of different destinations through time. This study provides the first application of a tourism climate index in the tropical southwest Indian Ocean, applying the recently developed Holiday Climate Index (HCI) for Réunion Island. The suitability of this index is evaluated for the case of this French department, with a particular focus on air conditioning availability in tourism accommodation establishments as this index excludes night-time thermal comfort. Both iterations of the HCI (HCI_Beach_ and HCI_Urban_) are computed with meteorological data from Roland Garros Airport for the period 1991–2020, exploring monthly, annual, and seasonal climatic suitability. Mean monthly HCI scores reveal considerable seasonality in climatic suitability for tourism on the island with scores ranging from 89.3 (‘excellent’) to 36.9 (‘marginal’) for the HCI_Beach_ and 85.0 (‘excellent’) to 27.5 (‘unacceptable’) for the HCI_Urban_, with more favourable scores calculated for July and August, displaying a clear austral winter peak seasonal classification. Over the 30-year period, there is no statically significant change in mean annual climatic suitability, and at a monthly scale, only one month of the year for each index displays statistically significant trends. These results are important in informing tourism strategies for the island to maximise visitor satisfaction through targeting advertising more deliberately for peak touristic climate suitability during the winter months.

**Supplementary Information:**

The online version contains supplementary material available at 10.1007/s00484-024-02700-x.

## Introduction

Tourism destinations can be multifaceted in their offerings and attractions. Climate is an important factor that impacts the selection of destination, availability of activities and overall satisfaction with the tourism experience (Gössling et al. 2012). Over the last four decades, several indices have been developed to quantify the climatic suitability of tourism activities and destinations (Mieczkowski 1985; Dubois et al. 2016; Matthews et al. 2021). From the initial and most widely applied Tourism Climate Index (TCI; Mieczkowski 1985) developed for general world tourism, more sector-specific indices have been developed over the past decade (de Freitas et al. 2008), including the Holiday Climate Index (HCI) for beach (Rutty et al. 2020) and urban tourism (Scott et al. 2016), the Camping Climate Index (CCI; Ma et al. 2020), and the Ski Climate Index (SCI; Demiroglu et al. 2021). The Holiday Climate Index (HCI) was designed to identify the climatic suitability of leisure tourism and to account for specific climate preferences of beach and urban tourism offerings of destinations, with the HCI_Urban_ developed from questionnaires administered to tourists across six European cities (Scott et al. 2016; Rutty et al. 2020). The primary difference between the TCI and the HCI is the removal of night-time thermal comfort due to the ubiquity of air conditioning in European cities (Scott et al. 2016: 85). While this has limited the use of the HCI in southern African countries where air conditioning is less common, and where electricity outages hinder the use of any air conditioning (Fitchett and Hoogendoorn 2018; Mushawemhuka et al. 2021; Noome and Fitchett 2022), a French department in the tropical southern Hemisphere provides a unique setting for the application of this index.

Réunion Island is one of five overseas French departments that are part of the European Union and, while governed by locally elected representatives, follow the same laws, regulations, building standards and many cultural norms as found in mainland France (Lacassagne 2017; Kołodzejski 2018). The island is located in the southwest Indian Ocean, between Madagascar and Mauritius. While the impacts of climate on tourism have been widely studied on the southern African subcontinent, very little research has been conducted for these tropical southwest Indian Ocean Islands (Saarinen et al. 2022). TripAdvisor reviews of this region have revealed tourist sensitivity to climate, particularly around precipitation, heat, and strong winds where a necessity for accurate information to allow for tourist preparedness is required (Fitchett et al. 2020). These islands face compounded climate change threats from the gradual changes in weather conditions, to a heightened severity of extreme climate events including tropical cyclones and heatwaves, and inundation of coastal regions from sea level rise (Douglass and Cooper 2020). At present, many of these islands have tourism sectors that thrive on offering year-round tropical climates suitable for sun, sea and surf (Fitchett et al. 2020; Saarinen et al. 2022).

Tourism climate index data is beneficial in providing the information that enables tourism sectors to effectively promote tourism destinations for the time of the year of most optimal climatic conditions, and to quantify changes in climatic suitability of a destination over time. Such indices have been used to determine changes in climatic suitability over recent decades (Mihăilă and Bistricean 2018; Alonso-Pérez et al. 2021) and identify future tourism climate resource projections (Scott and McBoyle 2001; Scott et al. 2004; Amelung and Viner 2006; Perch-Nielsen et al. 2010; Kubokawa et al. 2014; Fang and Yin 2015; Nguyen et al. 2020; El-Masry et al. 2022; Hidayat 2022; Li and Chen 2023). These indices have also identified trends in changing climate for tourism (Zhong et al. 2019; Samarasinghe et al. 2023) and been used to predict tourism arrivals to destinations (Aygün Oğur and Baycan 2022). Data from climate indices is pivotal in assisting tourism marketers and planners to strategise destination image and to plan offerings around off and peak periods (Mahmoud et al. 2019). With limited information of this nature available, this paper presents the first calculation of climatic suitability for the southwest Indian Ocean (SWIO) applying the HCI to Réunion Island for the period 1991–2020.

## Materials and methods

### Study site

**Fig. 1:**
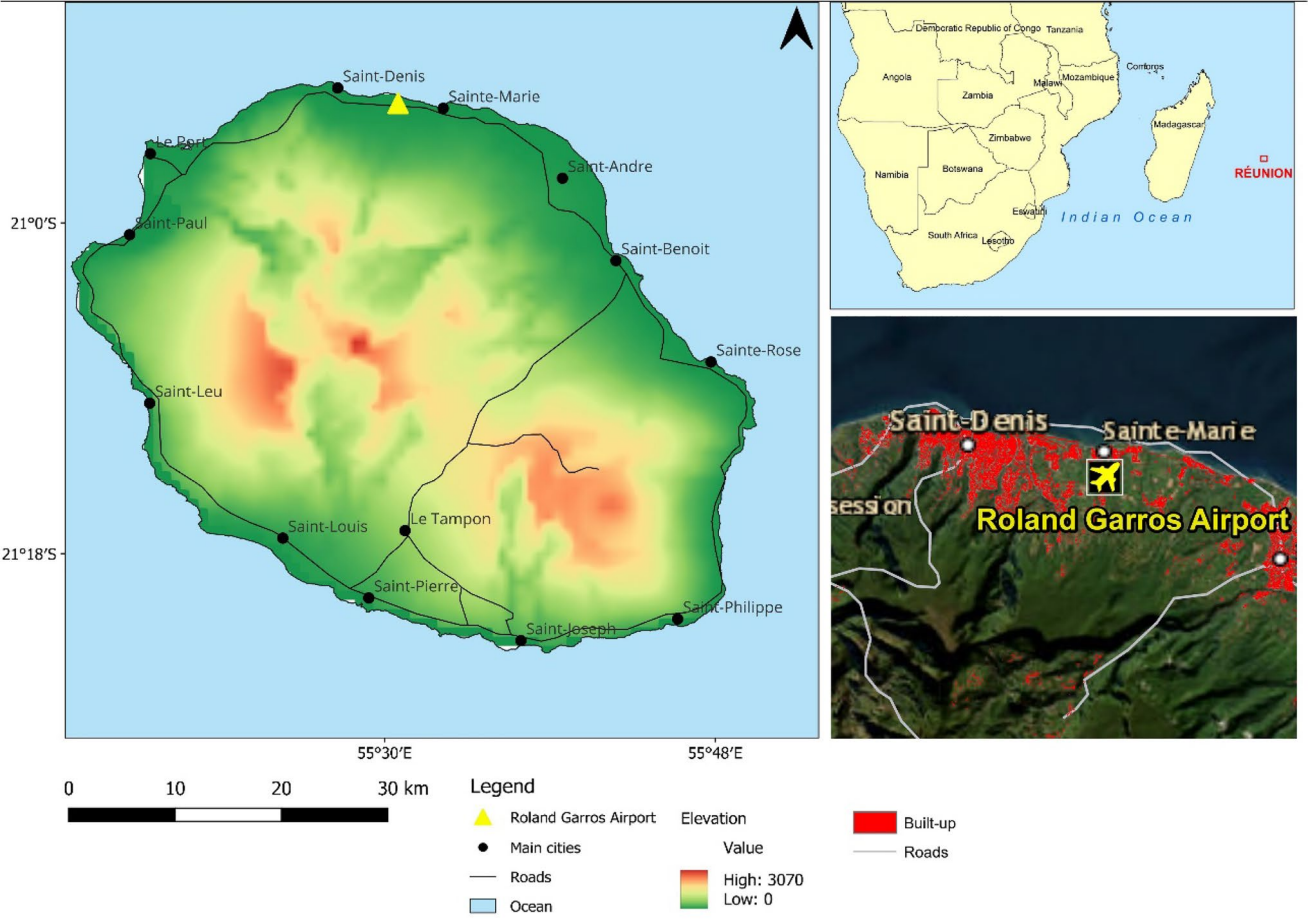
Location of study site, Roland Garros Airport, in Réunion Island

Located 680 km east of Madagascar and 170 km southwest of Mauritius, Réunion Island is a tropical island located in the southwest Indian Ocean (Bourjon and Fricke 2019). The island has a land area of 2,512km^2^ and is situated between 20°52ʹ-21°23ʹS and 55°13ʹ- 55°50ʹE with an altitude ranging from sea level to 3,071 m.asl (Bigot et al. 2019). Along with Rodrigues and Mauritius, the island forms part of the Mascarene archipelago (Bigot et al. 2019). Réunion is a volcanic island, formed two to three million years ago, and was occupied by France in 1663, becoming a French department in 1946 (La Réunion 2023). Since 1815, sugar cane, and in 1841, bourbon vanilla, have become key agricultural resources to the economy along with commerce, information technology, communications, and tourism (La Réunion 2023). Tourism accommodation establishments and touristic attractions are largely confined to the urban and coastal regions, as the rugged topography makes access to higher altitude regions difficult. Réunion Island offers attractions in cities that range from rum tasting, markets, urban parks, and several museum exhibitions centred around its history, and many host popular beaches, including Plage de L’Hermitage, Plage de La Saline les Bains, and Plage de Grande Anse (TripAdvisor 2023). Due to the size of the island, urban and beach tourism can be found in close proximity, and due to this potential overlap of experiences, both iterations of the HCI are useful to apply. Roland Garros Airport, the site for the meteorological data used in this study, is located in the coastal region of Sainte-Marie, 6 km from the centre of Saint-Denis the largest city in Réunion, along the northern coastline of the island, 2.5 km from the nearest beach and 10 km to the city centre that hosts an array of museums, cafés and street markets (Fig. 1).

The island is characterised by a warm tropical climate throughout the year and, due to its topography, experiences an elevation-controlled thermal gradient of 12–24 °C annual mean temperature (Vaxelaire 2018; Garot et al. 2019). Réunion Island experiences 1000–10,000 mm rainfall per annum, with greater precipitation on the east coast compared to the west coast (Vaxelaire 2018; Garot et al. 2019). Due to its location in the southwest Indian Ocean, the island is also impacted by tropical cyclones between November and April (Mavume et al. 2009), with individual storms contributing in excess of 4,000 mm of rain within the few days of their passage (Vaxelaire 2018).

### Data availability, suitability and validation

The use of the HCI in this study was validated through determining data availability, index suitability and tourist preferences (Fitchett and Meyer 2023). Daily resolution meteorological data (Fig. 1; 20°53ʹ28ʹʹS, 55°30ʹ45ʹʹE) was acquired from the Réunion Meteorological Services for a 30-year period spanning 1991–2020. There are a total of 11 meteorological stations on Réunion Island. A preliminary assessment of data availability found only two out of the 11 stations containing cloud cover data, Roland Garros Airport for the 30-year period (1991–2020) and Pierrefonds Airport with data spanning five years (2016–2020). Limited availability of climate data, particularly cloud cover and sunshine hours is a common occurrence in the southern hemisphere and Africa in particular (Fitchett et al. 2016; Noome and Fitchett 2019; Mushawemhuka et al. 2021). Data included minimum and maximum temperature (°C), maximum and mean humidity (%), precipitation (mm), wind speed (km/h), sunshine hours (hrs) and cloud cover (%). Data for the Roland Garros Airport study site was predominantly complete (99.5%); with a few instances of missing data for daily wind speed (0.5%), humidity (0.5%) and cloud cover (1.5%) over the complete 30-year period. To account for these gaps in data, triangulated daily, weekly, and annual averages were used as a form of proxy data.

To determine the suitability of the HCI in the context of Réunion Island, the innate assumption of air conditioning availability in accommodation establishments was examined. Popular booking sites of Booking.com, TripAdvisor, Kayak, Hotels.com and Trivago were identified; for each website, the total number of accommodation establishments available in Réunion Island was identified for flexible input dates. Using a filter function specific to each site, the amenity of air conditioning was searched for, providing a total of accommodations that also offer air conditioning, from which the proportion of all establishments listed on each site was calculated (Prinsloo and Fitchett 2023). Tourism preferences for the HCI have been investigated in previous work of Scott et al. (2016) who gathered tourism perception data from six European cities including Paris, France. Given that the island is a French department where the majority of tourists who visit are French (Statista 2023), we argue tourism preferences have already been validated for the island (Scott et al. 2016).

#### Index Calculation

As Réunion Island offers urban and beach tourism, both iterations of the HCI were applied. The HCI comprises three components considered important for leisure tourism, namely Thermal Comfort (TC) that combines daily maximum temperature (°C) and mean relative humidity (%); an Aesthetic (A) component as described by a percentage of cloud cover, and Physical component that combines Precipitation (P; mm) and Wind speed (W; km/h). Both iterations of the HCI consider the same three components, however the thermal comfort and aesthetic components are weighted differently (Eq. 1 and 2) due to the preferences of tourists in each setting, as tested for Europe and the Caribbean for urban and beach settings respectively (Scott et al. 2016; Rutty et al. 2020).

1 $$HC{I}_{Beach}=2\left(TC\right)+4\left(A\right)+\left(3\left(P\right)+W\right)$$

2 $$HC{I}_{Urban}=4\left(TC\right)+2\left(A\right)+\left(3\left(P\right)+W\right)$$

The above variables were converted to the requisite units and effective temperature, representing thermal comfort, was calculated using Humidex (Rutty et al. 2020; Matthews et al. 2021). Each variable was then rated on a scale of 0–10 with values input into both equations, resulting in a calculated output score from 0–100 (Scott et al. 2016; S1).

#### Data Analysis

Daily HCI_Beach_ and HCI_Urban_ scores were computed, and mean monthly scores averaged from these were classified for each month from 1991–2020. Output values could range from 0–100 where values ranging from 0–9 are classified as ‘dangerous’ as the lowest descriptive rating, and 90–100, classified as ‘ideal’ and the highest descriptive rating for this index (Scott et al. 2016; S2). Mean annual HCI scores for both the HCI_Beach_ and HCI_Urban_ were then calculated and classified according to the same descriptive rating (Scott et al. 2016; S2). Following Scott and McBoyle’s (2001) six seasonal classifications for tourism climate resources, mean monthly scores for the HCI were plotted to determine the period of peak climatic suitability for tourism. Change in suitability scores over the time period was then calculated using linear regression (Fitchett et al. 2017).

## Results

### Suitability of the HCI: Airconditioning Availability in Tourism Accommodation

Exploring the ubiquity of air conditioning from online listings of accommodation establishments revealed considerable variation per booking site. Of the 885 accommodation establishments listed on Kayak, 73.9% indicated that air conditioning was available (Table 1). By contrast, of the 186 hotels listed on Hotels.com, only 56.7% of listings had air conditioning (Table 1). Booking.com had the largest number of listings, at 2,694, of which 69.2% listed air conditioning (Table 1). While this may not be a comprehensive listing of all accommodations in Réunion Island, nor an absolute record of how widespread air conditioning is within accommodation establishments, it is indicative of more widespread availability of air conditioning than in much of the southern African subcontinent (Mushawemhuka et al. 2021; Noome and Fitchett 2022). The considerably more stable electrical grid in Réunion further means that available air conditioning would be able to be used at all times (Selosse et al. 2018). While 69.2% of accommodation establishments offering air conditioning is considerably lower than a ubiquitous availability, it is arguably sufficient to warrant the use of the HCI, particularly due to the low diurnal temperature range on a tropical island.

**Table 1:** Accommodation booking sites reveal total listings available, total listings and percentage of listings containing air conditioning

Accommodation site	Total listed	Total (Air conditioning)	%
Booking.com	2,694	1,808	69.2
TripAdvisor	644	57	8.9
Kayak	885	654	73.9
Hotels.com	187	106	56.7
Trivago	216	140	64.8

### Climate of Réunion

The mean annual climate for Réunion Island is a tropical savanna climate (Aw) according to Köppen-Geiger classification (Climate-data Organization 2023). The mean climate for the island was determined using Roland Garros Airport meteorological station, however, we do note the presence of microclimates across the island, particularly including the effect of altitude-related lapse rates. Higher resolution climate data collected from ground-based fieldwork would be beneficial in determining microclimates across the island but goes beyond the scope of the study. The warmest months throughout the 30-year period are January and February with a mean monthly maximum temperature of 30.2 °C with the lowest mean monthly maximum temperature of 25.4 °C seen in July (Table 2). The coldest months are July and August with a mean monthly minimum temperature of 18.2 °C (Table 2). The mean annual maximum temperature is 27.9 °C with a minimum temperature of 21.0 °C (Table 2). Réunion experiences high rainfall in the months of January and February consistent with the tropical cyclone season and lower rainfall in September and October. Mean monthly relative humidity is comparatively consistent throughout the year with higher humidity seen in the months of February (76.5%) and March (76.1%; Table 2). Wind speed is relatively high throughout the year with an average of 20.4 km/h (Table 2). Cloud cover is consistent throughout the year with an annual average of 50.2% of cloud cover recorded (Table 2).

**Table 2:** Mean monthly climatic variables for Réunion Island for 1991–2020

	Tmax (°C)	Tmin (°C)	R (mm)	W (km/h)	RH (%)	CC (%)
Jan	30.2	23.6	270.2	19.7	75.5	59.4
Feb	30.2	23.7	279.3	19.2	76.5	57.8
Mar	30.0	23.3	264.5	19.9	76.1	54.1
Apr	29.2	22.3	133.4	19.5	74.4	49.5
May	27.6	20.7	95.7	19.2	72.6	47.8
Jun	26.3	19.2	73.0	20.6	70.2	44.3
Jul	25.4	18.2	50.9	22.4	69.2	44.7
Aug	25.5	18.2	54.8	22.6	68.5	46.6
Sep	26.0	18.7	48.5	22.2	68.8	46.2
Oct	27.0	19.9	43.3	21.2	69.8	49.4
Nov	28.3	21.1	62.5	19.7	70.6	49.8
Dec	29.5	22.7	160.2	19.1	72.4	52.5
AVG	27.9	21.0	128.0	20.4	72.1	50.2

*W* Wind speed, *R* Rainfall, *RH* Relative humidity, *CC* Cloud cover

### HCI Scores

Mean annual scores for Roland Garros Airport for the HCI_Beach_ range from 66.5 (‘good’, 1998) to 72.3 (‘very good’, 1992) and HCI_Urban_ scores range from 55.2 (‘acceptable’, 1998) to 60.1 (‘good’, 1994) over the period 1991–2020 (Table 3). Overall, the annual average of HCI_Beach_ scores (69.6) are slightly higher than HCI_Urban_ scores (57.3) with a difference of 12.3 units. There is no clear pattern between periods of higher and lower mean annual scores. Within both iterations the climatic suitability ranges from ‘acceptable’ to ‘very good’.

**Table 3:**
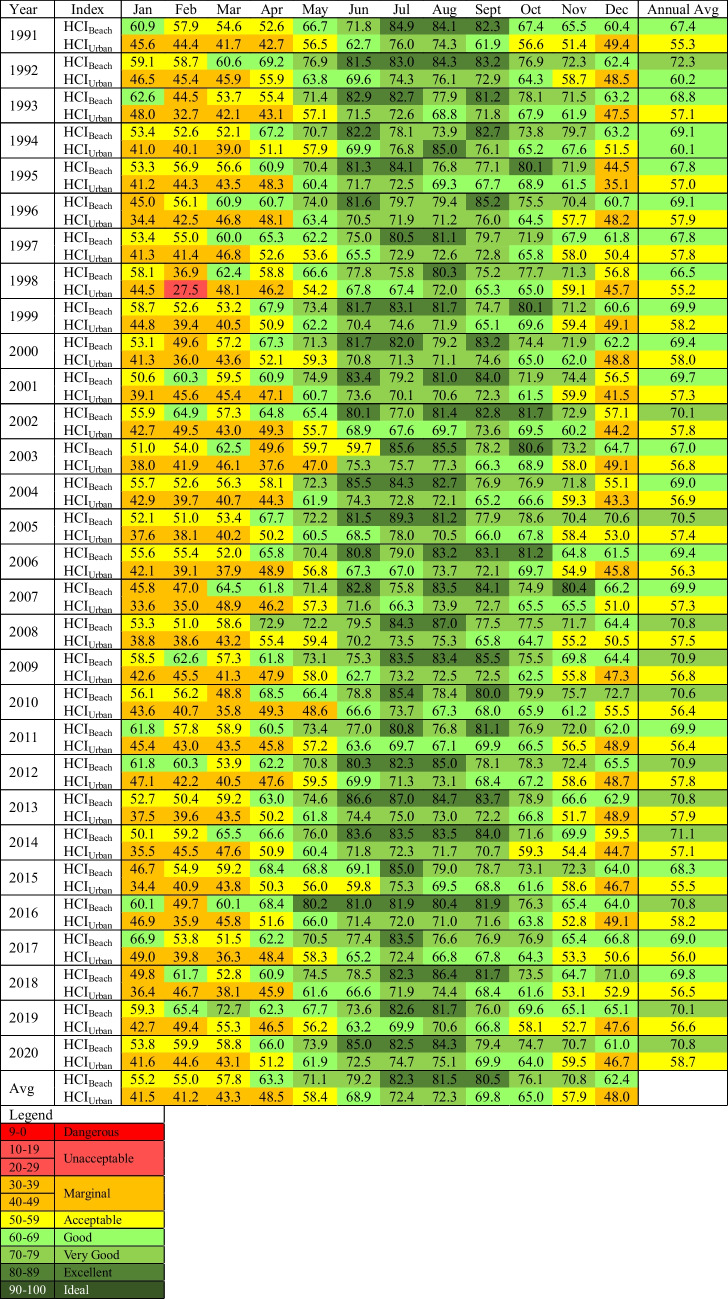
Mean monthly climate suitability scores for the HCI_Beach_ and HCI_Urban_ for Roland Garros Airport from 1991–2020

**Table 4:**
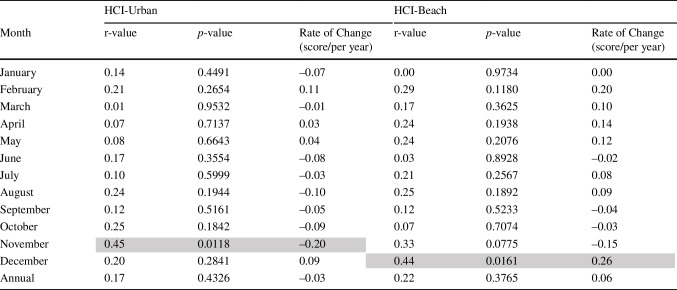
Regression analysis for HCI scores from Roland Garros Airport, 1991–2020

Exploring scores at a more granular monthly scale, for the period 1991–2020, mean monthly HCI_Beach_ scores range from 36.9 (‘marginal’) in February 1998 to 89.3 (‘excellent’) in July 2005. Mean monthly scores for HCI_Urban_ range from 27.5 (‘unacceptable’) in February 1998 to 85.0 (‘excellent’) in August 1994. For the HCI_Urban_, the lowest score is also the only classification of ‘unacceptable’ conditions at a monthly scale over the 30-year period. Further, both lowest scores for the HCI were found in the same month and year, February of 1998. Higher HCI scores are calculated for the months of July and August for both iterations of the index. Similar to mean annual scores, the HCI_Beach_ exhibits higher scores overall compared to the HCI_Urban_. The HCI_Beach_ reveals monthly scores classified as ‘excellent’ (23.9%), ‘very good’ (29.7%), ‘good’ (23.6%), ‘acceptable’ (19.4%), ‘marginal’ (3.3%) with no ‘unacceptable’ or ‘dangerous’ classifications. HCI_Urban_ monthly scores reveal classifications of ‘marginal’ (36.1%), ‘very good’ (20.6%), ‘good’ (25.0%), ‘acceptable’ (17.7%) and ‘excellent’ (0.3%) and ‘unacceptable’ (0.3%) over the 30-year period.

**Fig. 2:**
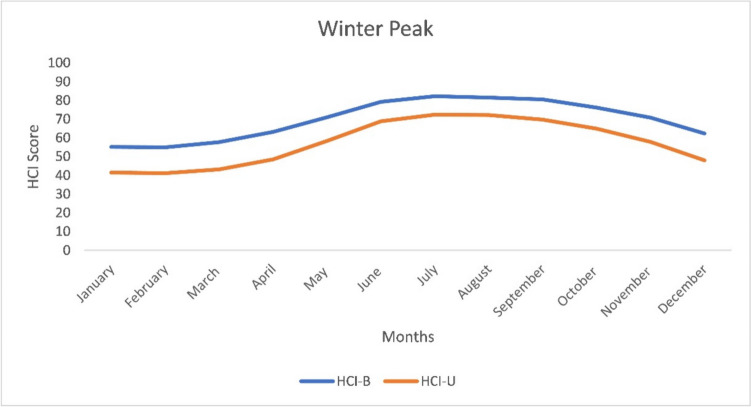
Seasonality of climate variability of Roland Garros Airport meteorological station is characterised by a late winter peak distribution

Following Scott and McBoyle’s (2001) six seasonal classifications, HCI scores for both HCI_Urban_ and HCI_Beach_ reveal a relatively late winter peak, with comparatively higher climatic suitability scores from June to September (Fig. 2). Lower scores in summer are a result of higher rainfall during these months, particularly during tropical cyclone season, and due to higher temperatures that exceed the threshold for thermal comfort. Higher scores in winter months result from cooler temperatures bringing thermal comfort into an ideal range coupled with lower rainfall totals. As the HCI does not explicitly consider the role of tropical cyclones, other than in elevating rainfall and windspeeds during their passage, this winter peak is fortuitous as it aligns with the period of very low tropical cyclone risk.

Calculating change in climatic suitability over time, there is no statistically significant change in mean annual index scores over the 30-year period for either the HCI_Beach_ or HCI_Urban_ (Table 4). Exploring the change in index scores for each month for HCI_Beach_ reveals a statistically significant increase in climatic suitability in December of 0.26 units per year (r = 0.44, p = 0.0161). HCI_Urban_ annual index scores reveal a statistically significant decrease in climatic suitability in November of 0.20 units per year (r = 0.45, p = 0.0118). Both of these occur during the period of lowest HCI scores, and the increase for December HCI_Beach_ scores is not at a rate sufficient to improve the climatic conditions to good or excellent levels. For the rest of the months for both HCI calculations, no statistically significant trends are calculated. This reveals a relatively static climatic suitability against which marketing decisions and policies can be made and implemented to maximise the tourist experience.

**Table 5:**
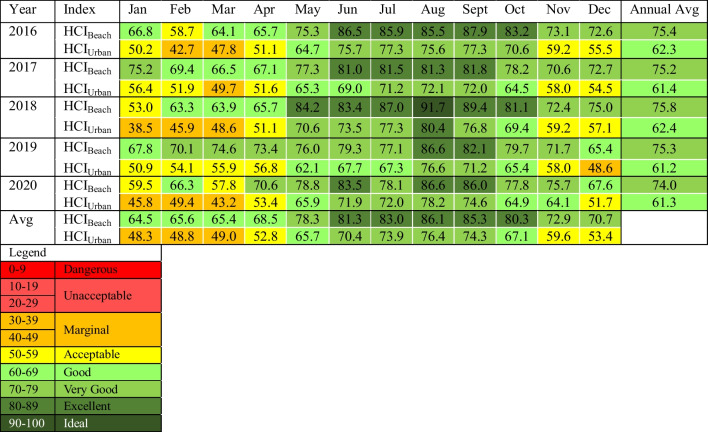
Mean monthly climate suitability scores for the HCI_Beach_ and HCI_Urban_ for Pierrefonds Airport from 2016-2020

The HCI_Beach_ and HCI_Urban_ were calculated for a 5-year period from 2016–2020 for Pierrefonds Airport (Table 5). Mean annual scores for the HCI_Beach_ range from 74.0 (‘very good’, 2020) to 75.8 (‘very good’, 2018) and HCI_Urban_ scores range from 61.2 (‘good’, 2019) to 62.4 (‘good’, 2018). It is noted that mean annual classifications for suitability remain the same for each index for the 5-year period. Mean monthly scores for the HCI_Beach_ range from 53.0 (‘acceptable’) in January 2018 to 91.7 (‘ideal’) in August 2018. For the HCI_Urban_, mean monthly scores range from 38.5 (‘marginal’) in January 2018 to 80.4 (‘excellent’) in August 2018. While long-term trends cannot be identified with Pierrefonds Airport meteorological data due to limited data, it is noted that mean annual HCI_Beach_ and HCI_Urban_ scores are marginally higher than Roland Garros Airport with scores increasing by an average of 5.04 units, classifying from ‘good’ to ‘very good’ for the HCI_Beach_ and scores increasing by 4.52 units, classifying from ‘acceptable’ to ‘good’ for the HCI_Urban_ (Table 3 and 5).

## Discussion

Tropical islands are often associated with touristic expectations of beautiful summer beach holidays characterised by sun, sand and surf weather (Mestanza-Ramón et al. 2020). Results from this study reveal that in Réunion this is not always the case. Over the 30-year period 1991–2020, no ‘ideal’ HCI_Beach_ or HCI_Urban_ scores were calculated at monthly or annual scale. Mean annual scores range from classifications of ‘acceptable’ to ‘very good’. Favourable climatic conditions are experienced during the winter season, reaching maximum scores classified as ‘very good’ to ‘excellent’. During the summer months, the climate is classified by the HCI_Urban_ and HCI_Beach_ as ranging from ‘acceptable’ to ‘marginal’, with occasional ‘unacceptable’ conditions. In many instances, these lower summer scores are due to the hot air temperatures, high relative humidity, and frequent cloud cover, and rainfall. These meteorological conditions would indeed detract from beach activities, and outdoor urban activities including urban parks, gardens and markets.

While this island does not provide year-round ideal climatic conditions, these results do reveal a climatic competitive advantage. The peak climatic conditions for tourism are experienced outside of the tropical cyclone season; while the island is not frequently affected by the landfall of tropical cyclones, it is located within the dominant storm track path (Fitchett and Grab 2014). As the South Indian Ocean is experiencing an increase in the frequency of category 5 tropical cyclones (Fitchett 2018), the size and impact of the storms in the southwest Indian Ocean is increasing, with resultant heavy rainfall, high wind speeds and storm surge events. In addition to these meteorological conditions being classified as ‘unsuitable’, and contributing to the lower scores in the months in which they occur, the impact on the tourism sector is more extreme than other ‘unsuitable’ conditions. Tropical cyclones often result in tourists being confined indoors for the duration of the storm in the interests of safety and can result in the grounding of planes, long delays in flights or cancelling of flights (Becken and Wilson 2013; Boyd 2023). Even relatively moderate tropical storms can result in temporary flooding and damage to beaches and tourism accommodation (Fitchett et al. 2016). Adverse weather occurring predominantly in an already unfavourable season allows for the climatic benefits of the winter season to be maximised.

The austral winter season provides two key touristic markets for Réunion Island to capitalise on. The first is European tourists, many of whom travel predominantly during their summer months when schools and universities have a long vacation (Amelung et al. 2007; Jacobs 2009). Long-haul flights are popular (Cohen and Higham 2011), and Réunion Island would offer a tropical island destination with favourable weather and familiar European culture. Compared to tropical destinations such as Sri Lanka (Samarasinghe et al. 2023) where HCI scores for beach attractions ranged from 45–57 (‘marginal’ to ‘acceptable’) and urban attractions ranged from 33–45 (‘marginal’) in May and 54–62 (‘marginal’, ‘acceptable’ and ‘good’) for HCI_Beach_ and 42–50 (‘marginal’ to ‘acceptable’) for HCI_Urban_ from June to August. Réunion offers comparatively more attractive beach climatic suitability during the same period with scores ranging from 59.7–89.3 (‘acceptable’ to ‘excellent’) and scores ranging from 47.0–85.0 (‘marginal’ to ‘excellent’) for urban attractions. The second market is tourists from the southern African subcontinent who would be experiencing cold winter conditions at this time of the year and may be seeking warmer weather without having to travel tens of thousands of kilometres in overnight flights. TCI scores calculated for southern Africa in Zimbabwe (Mushawemhuka et al. 2021) and Namibia (Noome and Fitchett 2022) reveal comparatively suitable scores for the winter period June to July with scores ranging respectively from 75.1–86.5 and 72–87 (‘very good’ to ‘excellent’ according to TCI rating categories), yet beaches and coastal waters are often too cold during these winter months for prolonged leisure. Réunion Island provides an alternative to outdoor tourism based on visits to national parks and safari experiences. Further, while a winter-season peak has been identified for many locations in southern Africa, TCI scores reveal less suitable scores in the winter period particularly in the coastal area of Cape Town, a popular tourism destination with scores ranging from 56.16–59.20 (‘acceptable’) due to increased winter rainfall and lower temperatures (Fitchett et al. 2017).

Effective use of this information derived from HCI calculations requires engagement with tourism operators, the tourism advertising for individual countries, and tourism policy makers. Many tourism destinations are impacted by a mild or extreme seasonality where strategising during off-season periods can reduce inefficient economic and natural resource use and potential profit loss (Lee et al. 2008; Cannas 2012). Given that climate experienced in winter periods in Réunion is more likely to provide tourist satisfaction than weather experienced in the summer season that could reduce perceived destination image, particularly if the effects of tropical cyclones produce unpleasant climatic experiences, marketing all year round may be less economically effective (Fitchett et al. 2020). Accurate information around touristic expectations of the weather is needed to add to tourist satisfaction of a holiday experience (Fitchett et al. 2020). Positive word of mouth and electronic word of mouth (e-WOM) through TripAdvisor reviews and social media, for example during the winter period can attract visitors, result in revisiting to Réunion and aid in destination loyalty, outweighing economic downturns during summer months (Phillips et al. 2013; Ramseook-Munhurrun et al. 2015; Nasar 2022). While the 30-year trends for Réunion reveal a largely stable climatic suitability, continued monitoring to determine changes to suitability for the island under contemporary climate change is imperative.

The value of tourism climate indices in informing decision-making does rely on the suitability of the index, and its internal assumptions, for the destination in question (Fitchett et al. 2017; Fitchett and Meyer 2023). The HCI was originally built on European tourist perspectives of climatic suitability, as evaluated from questionnaire responses (Scott et al. 2016). Given that 86% of tourists visiting Réunion Island are from France (Statista 2023), it is reasonable to assume that in these settings these assumptions hold true. However, it is worth noting that these European tourists’ perceptions regarding suitable climate were centred on providing feedback around European cities (Scott et al. 2016) rather than the islands of tropical islands. While the HCI_Beach_ has benefitted from validation in tropical contexts (Rutty and Scott 2013; Rutty et al. 2020), variable weightings that were predominantly informed by beach users in the Caribbean could benefit from greater responses and diversity in terms of the visiting population. The greatest consideration regarding the suitability of the HCI, is the removal of night-time thermal comfort from the original TCI (Mieczkowski 1985; Scott et al. 2016). While the analysis of web listings reveals that approximately 70% of accommodation establishments in Réunion have air conditioning, a better coverage than many southern African countries (Mushawemhuka et al. 2021; Noome and Fitchett 2022), availability is far from ubiquitous. Given the low diurnal temperature range in Réunion, relatively high night-time temperatures with potentially limited relief from air conditioning is a significant factor to consider. The quality of sleep when travelling has a large impact on the overall enjoyment and satisfaction of a tourist (Yang et al. 2021). Thus, the application of a wider suite of tourism climate indices in future may provide valuable insight into climatic suitability.

## Conclusion

Research output applying tourism climate indices is growing in the southern hemisphere, particularly in southern Africa. This study presents the first calculation of the HCI for beach and urban segments for Réunion Island and the southwest Indian Ocean. Réunion Island offers a destination image of tropical beach holidays along with many urban attractions to explore and is an ideal location for the application of both iterations of the HCI. As night-time thermal comfort is a component excluded from the HCI, the availability of air conditioning was explored, revealing that while most tourism accommodation establishments do have air conditioning and so for this study application of this index is argued to be suitable, this amenity is not completely ubiquitous and thus additional indices need to be investigated to determine further climatic suitability. In determining climatic suitability for the island, the use of higher resolution in-situ studies to address the effects of microclimates is also noted. HCI scores reveal that the winter months of June to August climate suitability is classified as ‘excellent’ for beach activities and ‘very good’ for urban activities. HCI_Beach_ scores were found to be comparatively higher than HCI_Urban_ scores by 12.6 units. This provides key opportunities for tourism outside of tropical cyclone season and the marketing around Réunion Island as a winter destination in the southern hemisphere and alternative summer destination in the northern hemisphere, particularly Europe should therefore be explored. Further, there is a need to translate climate suitability findings into tangible policy recommendations to provide relevant information for tourism decision-making processes for the island.

## Supplementary Information

Below is the link to the electronic supplementary material.

Supplementary file1 (DOCX 21 KB)

## Data Availability

Authors do not have the authority to share the data. Requests should be made directly to Meteo France Réunion office.
