# Publisher Correction: Quantifying climatic suitability for tourism in Southwest Indian Ocean Tropical Islands: Applying the Holiday Climate Index to Réunion Island

**DOI:** 10.1007/s00484-024-02713-6

**Published:** 2024-05-31

**Authors:** Ariel S. Prinsloo, Jennifer M. Fitchett

**Affiliations:** School of Geography, Archaeology and Environmental Studies, University of the Witwatersrand, Johannesburg, South Africa

**International Journal of Biometeorology**

10.1007/s00484-024-02700-x

The article was originally published with formatting errors associated with Tables 4, 5, and 6. Springer assumes responsibility for these errors and extends its apologies to the author group and readers of the article. The original article has been corrected.

