# Supplementary material for: Quantifying climatic suitability for tourism in Southwest Indian Ocean Tropical Islands: Applying the Holiday Climate Index to Réunion Island
Source: Int J Biometeorol. 2024 May 14;68(9):1717–28. doi: 10.1007/s00484-024-02700-x (PMC11461609; doi:10.1007/s00484-024-02700-x)
Supplement: Supplementary file 1 — Supplementary file1 (DOCX 21 KB) [file 484_2024_2700_MOESM1_ESM.docx]

Quantifying climatic suitability for tourism in Southwest Indian Ocean Tropical Islands: Applying the Holiday Climate Index to Réunion Island.

*International Journal of Biometeorology*

Ariel. S. Prinsloo and Jennifer. M. Fitchett*

School of Geography, Archaeology and Environmental Studies, University of the Witwatersrand, Johannesburg, South Africa.

*corresponding author: [Jennifer.Fitchett@wits.ac.za](mailto:Jennifer.Fitchett@wits.ac.za)

Supplementary Table 1: HCI ratings for Thermal Comfort (TC), Aesthetic (A) , Physical components with Precipitation (P) and wind (W) variables (adapted from Scott *et al.,* 2016; Rutty *et al.,* 2020; Matthews *et al.*, 2021).

| Rating | Thermal | | Aesthetic | | Physical | | | |
| --- | --- | --- | --- | --- | --- | --- | --- | --- |
|  | Thermal Comfort (°C) | | Cloud Cover (%) | | Precipitation (mm) | | Wind (km/h) | |
|  | HCI_Urban_ | HCI_Beach_ | HCI_Urban_ | HCI_Beach_ | HCI_Urban_ | HCI_Beach_ | HCI_Urban_ | HCI_Beach_ |
| -10 |  | ≤9.9 |  |  |  |  | ≥70.0 | ≥70.0 |
| -5 |  | 10.0-14.9 |  |  |  |  |  |  |
| -1 |  |  |  |  | ≥25.00 | ≥25.00 |  |  |
| 0 | ≥39.0 | ≥39.0;  15.0-16.9 |  |  | 12.00-24.99 | 12.00-24.99 | 50.0-69.9 | 50.0-69.9 |
| 1 | ≤ -6.0 | 17.0-17.9 | 100.0 |  |  |  |  |  |
| 2 | 37.0-38.9; -0.1- -5.9 | 38.0-38.9; 18.0-18.9 | 91.0-99.9 | ≥96.0 | 9.00-11.99 |  |  |  |
| 3 | 0-6.9 | 19.0-19.9 | 81.0-90.9 | 86.0-95.9 |  |  | 40.0-49.9 | 40.0-49.9 |
| 4 | 35.0-36.9; 7.0-10.9 | 37.0-37.9; 20.0-20.9 | 71.0-80.9 | 76.0-85.9 |  | 9.00-11.99 |  |  |
| 5 | 33.0-34.9; 11.0-14.9 | 36.0-36.9; 21.0-21.9 | 61.0-70.9 | 66.0-75.9 | 6.00-8.99 |  |  |  |
| 6 | 31.0-32.9; 15.0-17.9 | 35.0-35.9; 22.0-22.9 | 51.0-60.9 | 56.0-65.9 |  | 6.00-8.99 | 30.0-39.9 | 30.0-39.9 |
| 7 | 29.0-30.9; 18.0-19.9 | 34.0-34.9 | 41.0-50.9 | 46.0-55.9 |  |  |  |  |
| 8 | 27.0-28.9 | 33.0-33.9 | 0.0-0.9; 31.0-40.9 | 0-0.9;  36.0-45.9 | 3.00-5.99 | 3.0-5.99 | 0; 20.0-29.9 | 0-0.05; 20.0-29.9 |
| 9 | 26.0-26.9; 20.0-22.9 | 31.0-32.9; 26.0-27.9 | 1.0-10.9; 21.0-30.9 | 1.0-14.9; 26.0-35.9 | <3.00 | 0.01-2.99 | 10.0-19.9 | 10.0-19.9 |
| 10 | 23.0-25.9 | 28.0-30.9 | 11.0-20.9 | 15.0-25.9 | 0.00 | 0 | 0.1-9.9 | 0.6-9.9 |

Supplementary Table 1: HCI score descriptive categories (after Scott *et al.*, 2016).

| Descriptive Rating | |
| --- | --- |
| 9-0 | Dangerous |
| 10-19 | Unacceptable |
| 20-29 | Unacceptable |
| 30-39 | Marginal |
| 40-49 | Marginal |
| 50-59 | Acceptable |
| 60-69 | Good |
| 70-79 | Very Good |
| 80-89 | Excellent |
| 90-100 | Ideal |
